# Metastatic Esophageal Carcinoma Cells Exhibit Reduced Adhesion Strength and Enhanced Thermogenesis

**DOI:** 10.3390/cells10051213

**Published:** 2021-05-16

**Authors:** Zihe Huo, Mariana Sá Santos, Astrid Drenckhan, Stefan Holland-Cunz, Jakob R. Izbicki, Michael A. Nash, Stephanie J. Gros

**Affiliations:** 1 Department of Pediatric Surgery, University Children’s Hospital Basel, 4031 Basel, Switzerland; zihe.huo@ukbb.ch (Z.H.); Stefan.Holland-Cunz@ukbb.ch (S.H.-C.); 2 Department of Clinical Research, University of Basel, 4031 Basel, Switzerland; 3 Department of Chemistry, University of Basel, 4058 Basel, Switzerland; mariana.santos@unibas.ch (M.S.S.); michael.nash@unibas.ch (M.A.N.); 4 Department of Biosystems Science and Engineering, ETH Zurich, 4058 Basel, Switzerland; 5 Department of General, Visceral and Thoracic Surgery, University Medical Center Hamburg-Eppendorf, 20251 Hamburg, Germany; astrid.drenckhan@gmx.de (A.D.); izbicki@uke.de (J.R.I.)

**Keywords:** adenocarcinoma of the esophagus, micrometastasis, metastatic potential, tumor cell adhesion, isothermal microcalorimtery

## Abstract

Despite continuous improvements in multimodal therapeutic strategies, esophageal carcinoma maintains a high mortality rate. Metastases are a major life-limiting component; however, very little is known about why some tumors have high metastatic potential and others not. In this study, we investigated thermogenic activity and adhesion strength of primary tumor cells and corresponding metastatic cell lines derived from two patients with metastatic adenocarcinoma of the esophagus. We hypothesized that the increased metastatic potential of the metastatic cell lines correlates with higher thermogenic activity and decreased adhesion strength. Our data show that patient-derived metastatic esophageal tumor cells have a higher thermogenic profile as well as a decreased adhesion strength compared to their corresponding primary tumor cells. Using two paired esophageal carcinoma cell lines of primary tumor and lymph nodes makes the data unique. Both higher specific thermogenesis profile and decreased adhesion strength are associated with a higher metastatic potential. They are in congruence with the clinical patient presentation. Understanding these functional, biophysical properties of patient derived esophageal carcinoma cell lines will enable us to gain further insight into the mechanisms of metastatic potential of primary tumors and metastases. Microcalorimetric evaluation will furthermore allow for rapid assessment of new treatment options for primary tumor and metastases aimed at decreasing the metastatic potential.

## 1. Introduction

Many solid tumors are characterized by a good prognosis in early stages if a complete resection can be achieved but by a tremendously bad prognosis in metastatic stages. Metastases usually present the limitation of therapy and are associated with a high mortality rate. One of the cancer types in which this is highly pronounced is esophageal carcinoma, which shows an increasing incidence of adenocarcinoma in the western population over the past decades [1]. Mortality is mostly associated with metastatic spread. The question remains highly discussed as to what induces and assists tumor cells to migrate from the primary site to lymph nodes and distant organs. In this discussion, the concepts of invasion, extravasation, adhesion, tumor cell homing, selection of more aggressive cells within a heterogeneous tumor cell population, and influences of hypoxia on the primary tumor are of great importance. For example, we previously investigated the role of the hypoxia-inducible enzyme carbonic anhydrase IX (CAIX) in esophageal carcinoma and found that especially patients with a moderate CAIX expression have a higher metastatic rate, thus hypothesizing that tumor cells develop a higher metastatic potential in mild hypoxia [2]. This, however, is expected to present only one aspect of tumor cell dissemination. There are no universal molecular biomarkers to determine the risk of tumor cell dissemination in solid tumors.

An emerging alternative is the analysis of biophysical markers of tumor cells. The focus here lies on physical aspects of the cells themselves, such as cell deformability, cell membrane water transport, or adherence of cells to, for example, the extracellular matrix (ECM) [3,4,5,6]. Measuring adhesion of tumor cells to ECM proteins has become commonly used as a measure of metastatic potential of tumor cells [7,8]. There is experimental evidence for the validity of this relationship [9,10]. Using a spinning disc shear assay, it has been shown that in human breast cancer cells adhesion strength can potentially serve as a stable marker for migration and metastatic potential [9].

A further biophysical approach of tumor cell analysis is the investigation of the bioenergetic capability of tumor cells. This can, for example, be determined by measuring tumor thermogenesis, which in turn is linked with almost all cellular events in the cells’ energy turnover [11]. In this context, it has been proposed that metastatic cells have an enhanced thermogenesis. This was experimentally demonstrated for different cell lines with variations of metastatic potential including tongue squamous cell carcinoma, human non-small-cell lung carcinoma, human breast adeno carcinoma and murine melanoma [12]. However, primary and metastatic cells in these experiments did not originate from the same host. Experiments of these previous publications on the relationships of thermogenesis or adhesion strength to the metastatic potential of tumor cells used either cell lines of the same tumor entity but from different patients or artificial, metastatic subclones of the original cell line [10,12].

In the current study, we used two paired stable esophageal adenocarcinoma cell lines generated from primary tumors and lymph nodes of the same patients. Comparing cell lines derived from the same patient makes our data unique even though the number of patients is low, as matched primary tumor and lymph node cell lines are rare. Furthermore, we investigated the thermogenic activity as well as the adhesiveness of primary cells and their corresponding metastatic cell lines in combination the first time. We evaluated any correlations between metastatic potential of the metastatic cell lines and higher thermogenic activity or decreased cell adhesion strength, respectively.

## 2. Materials and Methods

### 2.1. Cell Lines and Culturing Conditions

Cell lines PT1590, LN1590, PT6216, LN6216c and LN6216gc were acquired from primary tumor and lymph nodes of patients with esophageal adenocarcinoma at the University Medical Center Hamburg-Eppendorf as described previously [13,14,15]. Cells were cultured in RPMI 1640 (Gibco, Thermo Fisher Scientific Inc., Waltham, MA, USA) with 10% fetal bovine serum (Gibco), 10 μmol/mL transferrin (Sigma-Aldrich, St. Louis, MO, USA), 1 μg/mL insulin (Sigma-Aldrich), 1 μg/mL fibroblast growth factor (Peprotech, Hamburg, Germany) and 1 μg/mL epidermal growth factor (Peprotech). This medium is abbreviated as TUM throughout the manuscript. All cells were cultured in a humidified atmosphere at 37 °C in air with 5% CO_2_.

### 2.2. Isothermal Microcalorimetry

For microcalorimetric measurements, a 48-channel isothermal microcalorimeter (calScreener, Symcel AB, Stockholm, Sweden) was used as previously described [16]. Tumor cells were seeded into vials and incubated for 6 h to attach in TUM medium. The vials were then sealed and inserted into the well-plate microcalorimeter according to manufacturers’ instructions. One position in the plate was loaded with an inert sample, which was used as a reference. For optimal performance, multiple separate reference vessels were included. Each reference vessel was filled with an inert sample (medium only), which was used as a thermal reference. Following thermal equilibration, measurements were recorded with the thermostat set at 37 °C. The microcalorimeter data were sampled at a frequency of 1 data point every 60 s over >250 h until the metabolic heat signal returned to baseline. Data were stored by the Symcel calView software and exported as a CVS file that could be edited in commonly used spreadsheet software. This assay was performed in triplicate and repeated. Data were analyzed using GraphPad Prism 8.4 software (GraphPad Software, San Diego, CA, USA). For testing of significance, the two-sided *t*-test or analysis of variance with a post-hoc test was used. *p* values less than 0.05 were defined as significant. The error bars in all bar plots represent one standard deviation.

### 2.3. Shear Stress Adhesion Assays

The spinning disk apparatus was built in-house based on previously described designs [17,18]. The apparatus consists of a buffer bath sitting on a laboratory jack and a programmable DC motor (D.C. Servo Motor, Dynetic Systems) supported by a rotatable stainless-steel arm. Cover glasses were secured onto the motor through vacuum suction provided by a line running through the central rotary shaft of the device. A 3D-printed adaptor made from methacrylate resin (FormLabs) with a hole in the center was designed to fix the inverted cover glass in place during spinning. In an effort to minimize bulk fluid rotation and avoid disrupting the laminar boundary layer, 3D-printed top and side baffles were used in the water bath and the cover glasses were maintained at a height of 25 mm from the bottom of the chamber during the adhesion assay. The 25 mm glass cover glasses (Menzel Glaser) were sonicated in 50% ethanol for 15 min, rinsed with water and incubated in piranha solution (1:1 H_2_SO_4_ (concentrated): H_2_O_2_ (30%) (*v*/*v*)) for 30 min. Cover glasses were washed in ultrapure water and incubated for 1 h with shaking in 2% *v*/*v* (3-Glycidoxypropyl) trimethoxysilane (ABCR GmbH, Karlsruhe, Germany) in a solution of 88% *v*/*v* ethanol and 10% *v*/*v* water. Subsequently, the surfaces were washed in ethanol and incubated at 80 °C for 30 min. Silanized cover glasses were then functionalized with 1 mg/mL Collagen type I (FlexiCol, Sigma) in 100 mM HEPES pH 8 for 1 h, washed with ultrapure water and dried with nitrogen. Then, the surface was blocked with 5% heat-inactivated BSA in PBS for 30 min and washed with ultrapure water. Esophageal carcinoma cells were allowed to attach for 4 h at 37 °C. Before spinning, the cell suspension was removed and replaced with PBS; then, the cover glasses were mounted on the spinning disk device and immersed in PBS at room temperature. The spinning routine consisted of a 20 s acceleration ramp, 5 min steady spinning at the indicated rotations per minute value, and 20 s deceleration. The hydrodynamic forces at any point on the surface of the cover glass vary linearly with radial distance and can be described by the following equation:

(1) τ=0.800r(ρµω3)1/2

τ is the applied shear stress, r is the radial position relative to the center of the cover glass, ρ is the spinning buffer density, µ is the fluid dynamic viscosity, and ω is the rotational speed. After spinning, the cover glasses were imaged at 10× magnification on an Olympus IX81 microscope (~500 individual images automatically stitched together with CellSens software (version 1.16, Olympus, Tokyo, Japan)). A custom Python-based image analysis script was used to segment the cells from the background by applying a gray-value threshold. The script returns the radius of the cover glass and a list containing the area, XY coordinates and distance to the center of the cover glass, for each cell. The fraction of adherent cells (f), at different positions on the disk, was calculated by normalizing the density of cells at each section of the disk with the density of cells at the center of the disk, where the shear forces are close to zero. Detachment profiles (f vs. τ) for three cell replicates per line were plotted and fitted with a global sigmoid probabilistic model to extract the value of the shear stress at which 50% of the cells remain adherent (τ_50_) [19]:

(2) $$f=1/\left(a-\exp\;\left[b\;\left(\tau -\tau_{50}\right)\right]\right)$$

The τ_50_ is used as a measure of mean adhesion strength for comparison of the different cell lines and is reported with 95% confidence interval determined by the fit.

## 3. Results

### 3.1. Origin of Cell Lines

PT1590 cell line was established from a patient with an apparently localized adenocarcinoma of the esophagus pT1, pN_1_, M0, G3 in 1997 [13]. The patient had undergone radical esophagectomy. The cell line LN1590 was generated from a lymph node of the same patient which had been classified by routine pathology (hematoxylin–eosin staining) as negative for metastases, but in which epithelial staining with Ber-Ep4-positive cells revealed micrometastases [13]. After culturing the cells, they had been subcutaneously transplanted into mice and led to progressive tumor development [13,20]. These findings reveal the aggressive potential of seemingly harmless and mostly undetected tumor cells. Since then, tumor classification has been modified to accommodate a more graduated differentiation of lymph node metastases in esophageal carcinoma.

**Figure 1:**
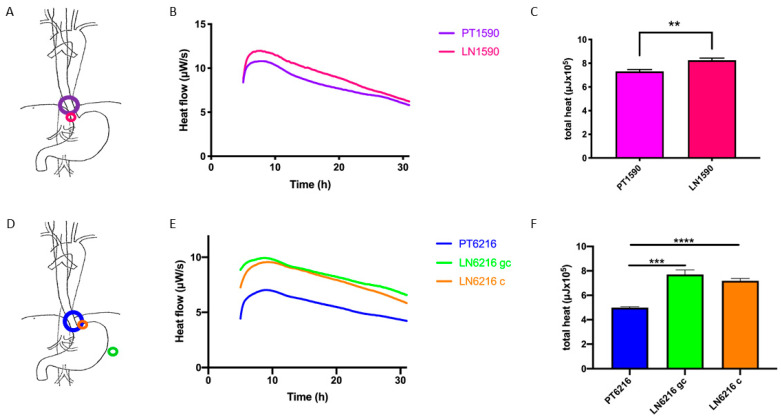
Esophageal carcinoma primary tumor and metastatic cells: location, microcalorimetric profile, and heat production. Cell lines PT1590 and LN1590 originated from the same patient with esophageal adenocarcinoma. LN1590 was derived from a micrometastic lymph node (**A**). Microcalorimetric assessments of tumor cells show an increase thermogenic profile of the metastasis compared to the primary tumor (**B**). This is also displayed in the increased overall heat production of metastatic cells. LN1590 cells have a significantly higher (*p* < 0.01 (**)) overall heat production (**C**). Cell lines PT6216, LN6216g, and LN6216c originated from primary tumor and lymph node metastases (LN6216gc, location of lymph node along the great curvature of the stomach; LN6216c, lymph node location at the cardia) of a patient with advance esophageal carcinoma with extensive metastatic spread (**D**). Microcalorimetry shows a higher heat flow rate for all metastatic cell lines compared to the primary tumor cell line (**E**). Both metastatic cell lines LN6216c and LN621gc have a significantly higher overall heat production (*p* < 0.0001 (****) and *p* < 0.001 (***), respectively) compared with the primary tumor cell line (**F**).

The human cell lines PT6216, LN6216c and LN6216gc were isolated from primary tumor and lymph node metastases of a patient with adenocarcinoma of the esophagus [15]. After initial staging uT3, N1, cM0, the patient received neoadjuvant chemotherapy. At the time of surgery, the tumor had progressed and was classified as pT4b, pN2, G3, L1, V1 (UICC IIIc) [15]. After R1-resection, the disease progressed rapidly despite of radiochemotherapy. PT6216 originated from the primary tumor, LN6216c from a metastasis of a lymph node localized at the cardia, and LN6216gc from a lymph node at the great curvature of the stomach (Figure 1A,D).

With respect to the location of the primary tumor at the distal esophagus, the closest lymph node from the tumor was LN6216c, and LN6216gc was further away.

### 3.2. Microcalorimteric Assessment of Tumor Cells

Isothermal microcalorimetry was performed to compare the heat production of cells from the primary tumor to that of tumor cells generated from lymph nodes from both patients separately. Cell line LN1590 showed an increased heat flow over 24 h as well as a much higher overall heat production compared with cell line PT1590 (Figure 1B,C). The peak heat flow value and total heat production increased around 10% and more than 20%, respectively, between PT1590 cell line and LN1590 cell line. The same phenomenon could be observed with cell lines derived from patient 6216. LN 6216c and LN6216gc showed much increased heat flow as well as a much higher heat total production (Figure 1E,F). The peak heat flow value and total heat production increased around 50% and more than 100%, respectively, between PT6216 cell line and corresponding metastatic cell lines. Interestingly, the lymph node with the highest activity is LN6216gc, which was located at the great curvature. 

### 3.3. Shear Stress Adhesion Assay

**Figure 2:**
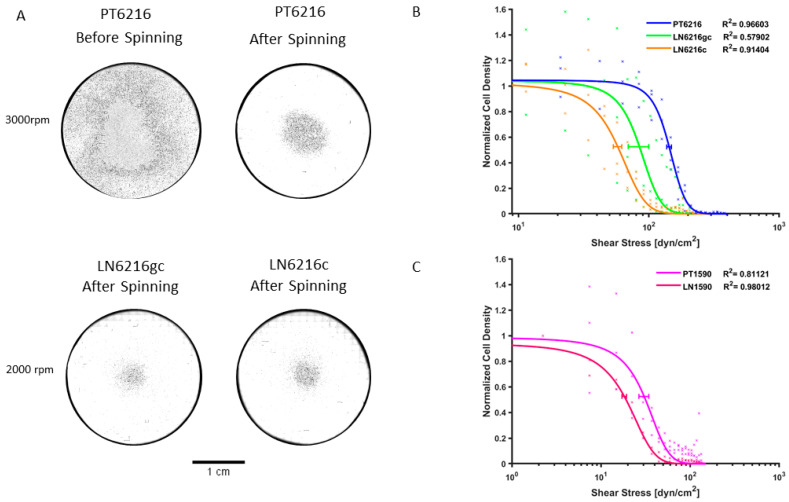
Adhesiveness of primary tumor and metastatic esophageal cells. We performed a shear stress adhesion assay. (**A**) The spinning disks seeded with PT6216 before and after spinning at 3000 rpm as well as disks with LN6216gc and LN6216c after spinning at 2000 rpm. The difference in rotational speed is due to the different adhesive properties of primary and lymph node cells. τ_50_ was calculated independently of this and reached a value of 143.28 ± 6.01 dyn/cm^2^ for PT6216 cell. τ_50_ was much lower for metastatic cell lines, 84.72 ± 15.04 dyn/cm^2^ for LN6216gc and 57.72 ± 4.26 dyn/cm^2^ for LN6216c cells. Less shear stress was necessary to detach LN6216gc and LN6216c cells in comparison to PT6216 cells. (**B**) Error bars represent the 95% confidence interval of τ_50_ calculated by the global fit. (**C**) τ_50_ was lower for the metastatic cell line LN1590 with 18.28 ± 1.10 dyn/cm^2^ than for primary tumor cell line PT1590 with 30.52 ± 3.81 dyn/cm^2^ cells. Less shear stress was necessary to detach LN1590 cells in comparison to PT1590 cells.

To analyze the adhesiveness of these esophageal carcinoma cell lines, we performed a shear stress adhesion assay comparing primary tumor cell line PT6216 with lymph node cell line LN6216gc and LN6216c. The τ_50_ parameter is the shear stress at which 50% of the cells are detached from the disk. τ_50_ for PT6216 was 143.28 ± 6.01 dyn/cm^2^, while it was 87.72 ± 15.04 dyn/cm^2^ for LN6216gc and 57.72 ± 4.26 dyn/cm^2^ for LN6216c. The assay revealed that, after 4 h of incubation, both LN6216gc and LN6216c cells are less adhesive compared with PT6216 cells, which results in a greater readiness to detach (Figure 2). Depending on the adhesion strength of the cell lines, we found that different rotational speeds could be used so that the detachment midpoint of the given cell line fell within a reasonable range for the assay. While both lymph node cell lines easily detached at 2000 rpm, PT6216 cells were optimally spun at 3000 rpm to sufficiently distribute cells across the disk and allow proper image analysis and data fitting. PT1590 and LN1590 were spun at 1500 rpm to achieve sufficient cell distribution and resulted in a τ_50_ for PT1590 of 30.52 ± 3.81 dyn/cm^2^ and 18.28 ± 1.10 dyn/cm^2^ for LN1590 cells.

## 4. Discussion

While the incidence of esophageal adenocarcinoma in the Western countries is continuously increasing, the 5-year mortality rate remains poor [1,21]. Clinically, multimodal therapy concepts are continuously reevaluated and improved. Nevertheless, mortality in patients with solid tumors is often associated with metastatic spread. In the light of this, scientific efforts should focus on understanding the metastatic process in order to stop or prevent metastases. Tumor cell dissemination is a highly complex process of which not all steps are fully understood yet. While some aspects such as tumor cell invasion, extravasation, migration, and homing have been identified, it remains unclear how these tumor cell attributes work together in actual cell dissemination. Mechanisms targeting specific cell attributes have been described, and new targeted as well as immuno-therapies are emerging. 

We previously identified HER2 and CXCR4 as possible alternative targets in the treatment of esophageal carcinoma and associated mild hypoxia leading to expression of CAIX with increased metastatic spread [2,22,23,24,25]. There is, however, no universal molecular marker on the basis of which metastases can be treated. More recently, biophysical cell attributes that contribute to tumor cell dissemination have become of interest. 

Our data show that patient-derived metastatic esophageal tumor cells have a higher thermogenic profile as well as a decreased adhesion strength compared to their corresponding primary tumor cells. Both the higher thermogenic profile and the decreased adhesion strength are associated with a higher metastatic potential. This also correlates with the clinical patient presentation. Matched patient derived stable cell lines are rare in any tumor disease, and these paired esophageal carcinoma cell lines derived from primary tumor and metastatic lymph nodes of the same patient are exceptional. Previous studies on the relationships of either thermogenesis or adhesion strength to the metastatic potential of tumor cells have used cell lines of the same tumor entity that had been derived from different patients or metastatic subclones that were established by in vitro sorting of the original cell line [10,12].

Future research will be dedicated to understanding the cellular mechanisms of these adverse functional, biophysical properties of potentially metastatic tumors cells in detail. Microcalorimetric evaluation will furthermore allow for rapid assessment of the metastatic potential as well as new treatment options for primary tumor and metastases.

### 4.1. Biophysical Assays to Determine Metastatic Potential

Tumor cells within one tumor are highly heterogeneous. It has been established that not all cell populations of one tumor are capable of forming metastases. The process of forming metastases depends on several important and highly orchestrated steps [26]. Furthermore, it has been shown that certain conditions such as tumor cell hypoxia can facilitate this process [27]. Studies on tumor cell adhesion have shown that, to migrate, cells have to turn over their focal adhesions in their interaction with the ECM [28]. Migration speed thus becomes a function of adhesion strength [29]. Any disruption of normal adhesion strength can lead to migration of otherwise stable cells. Invasive cancer cells express more dynamic focal adhesions and display a decreased adhesion strength [30,31]. There is evidence that adhesion strength can point to differences in the metastatic potential. Using the shear stress adhesion assay, biophysical properties of the cells can be utilized to anticipate the aggressiveness of tumor spread and even mark a subpopulation of metastatic cells [9].

A further biophysical aspect of tumor cell behavior is thermogenesis. Thermogenesis is a reflection of all cellular processes for which energy is necessary. Tumor cells for example exhibit a significantly different and elevated metabolism from that of other cells. Changes in lipid metabolism have been linked with changes of the thermogenic profile [12]. Thermogenesis can be measured by isothermal microcalorimetry. Several microcalorimetric devices have been deployed to measure changes in heat flow of bacteria, protozoan, and human cells [16]. Isothermal microcalorimetry has been used in drug screening in microbiology and monitoring in food microbiology and material testing as well as in the investigation of parasitological questions [16,32,33,34]. We recently showed the applicability of isothermal microcalorimetry in the rapid assessment of drug response in a rare and highly malignant case of pediatric clear cell sarcoma of the kidney [35]. Microcalorimetry is highly sensitive and measures in the range of microwatt under isothermal conditions [11]. Experimental conditions can be adapted to address specific scientific questions.

Our two adenocarcinoma cell lines originating from primary tumors and their metastatic counterparts are unique, as they present both primary and metastatic cell types of the same patient. Our data confirm that metastatic cells of both patients have a decreased shear adhesion strength combined with a higher thermogenesis and a clinically proven higher metastatic potential.

### 4.2. Origin and Localization of Metastases

All cell lines used in this study were derived from esophageal adenocarcinoma. However, patient characteristics and cell lines vary in several aspects. Of special interest is that LN1590 was generated from a micrometastasis in a lymph node that had been classified as tumor free by routine pathology in a previously untreated patient [13,20]. Nevertheless, injected into the flanks of immune-deficient mice, it led to aggressive tumor formation. At the time, these experimental findings revolutionized the perception of metastatic potential and clinical practice of esophageal carcinoma. Our data strongly support the notion that metastatic cells, even if derived from few metastatic cells in a seemingly intact lymph node, can display an aggressive phenotype with an increased thermo-energetic profile and a decreased adhesiveness. On the other hand, Patient 6216 had been treated with neo-adjuvant chemotherapy prior to resection but was presenting with poor response. In this case, we were able to derive stable cells from the primary tumor and two different lymph nodes located at the cardia and the great curvature, all of which were grossly tumor infiltrated. We observed a significantly increased thermogenic profile in all lymph nodes compared to the primary tumor. This raises a question that needs to be answered, whether the distance of the metastases from the primary tumor relates to the biophysical profile of the tumor cells. A microcalorimetric library comparing thermo-energetic profiles of different primary tumors and metastases might help to answer these questions in future experiments.

### 4.3. Possible Heat Generators and Sources of Thermogenesis in the Migrating Cell

Tumor cells display a reprogramed metabolism. It has been suggested that metastatic cells utilize metabolic pathways that supply extra energy to enable increased motility and invasiveness [12]. For example, Lemos et al. described an increased heat release exclusively in metastatic cell lines of different origins (human oral squamous cell carcinoma, melanoma, non-small-cell lung and breast cancer, and murine melanoma cells) [12]. They investigated the influence of MAGEA10 protein on this process. Silencing of MAGEA10 led to a reduction of heat release as well as a reduction of cell adhesion and increase of migration. They further investigated the influence of uncoupling proteins, mainly UCP, and suggested a possible mitochondrial contribution in metastatic cell thermogenesis by enhancing thermogenesis through mitochondrial uncoupling. There is little known about the mechanisms of this, but one explanation for higher thermogenesis of migrating tumor cells might result from an increased channeling of ATP from fatty acid oxidation [12].

One of the mechanisms of tumor cell metabolism is the metabolic reprograming by which the cells exploit different pathways for ATP retrieval such as glycolysis, the oxidative phosphorylation system, the pentose phosphate pathway, and others to generate ATP. Our group was recently able to show an increased thermogenesis in neuroblastoma cells that favor fructose over glucose metabolism, a metabolism that provides them with a survival benefit under unfavorable low oxygen and low nutrient supply when fructose is available (Pini et al., submitted). These same unfavorable conditions are also known to promote metastasis.

The factors contributing to tumor cell adhesion are heterogeneous and complex. Several adhesion factors have been proposed to play a role in metastatic processes. For example, it has been shown that with a decrease of adhesion strength tumor cells display a decreased focal adhesion turnover [10]. Moreover, there are diverting reports on the role of integrin activation and its role in metastatic behavior. Analysis of genes upregulated in weakly adherent cells include significant enrichment of genes involved in microtubule and cytoskeletal components as well as increased FA turnover and migration, motor proteins, and cytoskeletal contraction [5]. Indeed, metastatic cells have been found to be more contractile [5,36,37].

**Figure 3:**
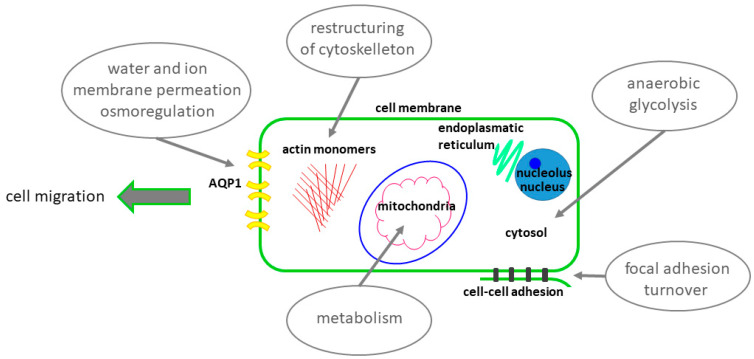
Possible heat generators and sources of thermogenesis in the migrating cell. Schematic overview of cellular processes that might contribute to increased thermogenesis of the migrating cell such as water and ion membrane permeation, osmoregulation, restructuring if cytoskelleton, anaerobic glycolysis, focal adhesion turnover or mitochondrial metabolism.

Our own investigations in migrating neuroblastoma cells have revealed an increased expression of the water channel AQP1 that furthers migration by promoting water in- and efflux through the tumor cell membrane and facilitating restructuring of the cytoskeleton [38]. On the other hand, the inhibition of the polymerization of actin led to a reduced heat release [12]. Increased expression of water channels is followed by increased efforts of the cell to sustain osmoregulation. These processes require higher energy levels. A schematic overview of possible heat generators and sources of thermogenesis in the migrating cell is summarized in Figure 3.

Our future investigation will focus on the role of AQP1-facilitated membrane permeability and plasticity and possible role in tumor cell thermogenesis for the migratory process.

### 4.4. Clinical Implications

In health, cell migration is a highly orchestrated process that is required for embryonic development. It is vital for tissue repair and regeneration [28,39]. In cancer, cell migration determines the severity of the disease and its metastatic potential. The metastatic potential of a specific patient’s tumor can be determined most clearly in hindsight. However, when metastases are seen radiographically, it might already be too late for a successful treatment. Are there factors that can allow us to determine the metastatic potential a priori? We previously showed that we can assess response to alternative treatment approaches by using isothermal microcalorimetry of tumor slice cultures [35]. Hypothesizing that each tumor and each metastasis has a specific microcalorimetric profile and cell adhesiveness contributing to its metastatic potential, a systematic compilation of these data could enable us to predict the metastatic potential and thus the aggressiveness of tumor disease. Furthermore, microcalorimetric evaluation allows rapid assessment of treatment response of both primary and metastatic tissues. This will increase the possibilities for investigating treatment of metastases and the metastatic capabilities in addition to those of the primary tumor.

## Data Availability

The datasets generate for this study are available on request from the corresponding author.
